# The p70S6K Specific Inhibitor PF-4708671 Impedes Non-Small Cell Lung Cancer Growth

**DOI:** 10.1371/journal.pone.0147185

**Published:** 2016-01-15

**Authors:** Zhi-Xin Qiu, Rong-Fei Sun, Xian-Ming Mo, Wei-Min Li

**Affiliations:** 1 Department of Respiratory Medicine, West China Hospital, Sichuan University, Chengdu, 610041, China; 2 Department of Respiratory Medicine, Tianjin First Central Hospital, Tianjin, 300192, China; 3 Laboratory of Stem Cell Biology, State Key Laboratory of Biotherapy, West China Hospital, Sichuan University, Chengdu, 610041, China; University of Navarra, SPAIN

## Abstract

**Background:**

As a serine/threonine protein kinase, p70S6K plays an important role in tumor cells. Evidence has revealed overexpression of p70S6K and phosphorylated p70S6K (p-p70S6K) in various tumor tissues, with these proteins identified as independent prognostic markers in non-small cell lung cancer (NSCLC). In this study, we explored the role of the p70S6K specific inhibitor PF-4708671 in NSCLC.

**Methods:**

Three NSCLC cell lines (A549, SK-MES-1, and NCI-H460) were treated with PF-4708671 at five different concentrations, including 0.1μM, 0.3μM, 1μM, 3μM and 10μM, and protein levels were determined by Western-blot. Then, PF-4708671’s effects were assessed both *in vitro* (cell proliferation, apoptosis, cell cycle distribution, and invasion) and *in vivo*.

**Results:**

The expression levels of p-p70S6K and the downstream effector S6 were significantly reduced by PF-4708671. Diametrically opposite, the downstream protein levels of BAD, Caspase3 and ERK had increased after treatment with PF-4708671. In addition, PF-4708671 drastically inhibited cell proliferation and invasion ability in A549, SK-MES-1 and NCI-H460 cells *in vitro*, causing cell cycle arrest in G0-G1 phase. Limited effects of PF-4708671 were observed on apoptosis in the three NSCLC cell lines assessed. Importantly, PF-4708671 could inhibit tumorigenesis in nude mice *in vivo*.

**Conclusion:**

These findings demonstrated that the p70S6K specific inhibitor PF-4708671 has inhibitory effects on NSCLC tumorigenesis *in vitro* and *in vivo*. Therefore, P70S6K should be considered a new potential therapeutic target, and PF-470867 may be used as targeted drug for cancer treatment.

## Background

Lung cancer, one of the most common malignancies, is the leading cause of cancer-related deaths worldwide, with the highest morbidity and mortality rates in China. More than 80% of patients with lung cancer are diagnosed as non-small cell lung cancer [1]. Despite combination therapy with surgical resection, chemotherapy, radiotherapy, and biological target therapy, the overall 5-year survival rate of lung cancer is only 16%, varying from 52.2% to 4% in local and distant stage patients, respectively [2]. Therefore, identification of novel targets and elucidation of changes at the molecular level could be beneficial to lung cancer treatment.

As a serine/threonine protein kinase of the AGC kinase family, p70S6K is phosphorylated by different growth factors and insulin-like factors through the PI3K/mTOR pathway, and interacts with S6, eIF4B, eEF2K, PDCD4 and many other substrates; this is important for mitogen-induced cell proliferation, survival, motility and chemotherapy drug resistance in cancer cells [3]. Recent studies demonstrated that overexpression of p70S6K and p-p70S6K in various tumor tissues is important in predicting poor prognosis, and contributes to chemotherapy resistance [4–14]. *In vitro* overexpression of p70S6K promotes cell proliferation, angiogenesis, and apoptosis suppression [15, 16]. Meanwhile, S6K1 knockdown inhibits p70S6K expression and significantly reduces cell proliferation, decreasing tumor formation in nude mice [17]. Moreover, p70S6K and p-p70S6K levels are significantly higher in tumors than in normal tissues from NSCLC patients [18, 19]. Our previous study showed that p-p70S6K is closely related to long-term survival in NSCLC [20]. Therefore, p70S6K plays an important role in NSCLC, and assessment of high specificity p70S6K inhibitors could help further define this new therapeutic target for clinical application.

Current studies of target therapies for cancer mostly focus on mTOR inhibitors [20], while works specifically assessing p70S6K inhibitors in lung cancer treatment are limited [21–25]. This study focused on the specific p70S6K inhibitor PF–4708671 to assess the effects of p70S6K inhibition in NSCLC [22].

## Methods

### 1. PF–4708671 (C_19_H_21_F_3_N_6_)

PF–4708671(#559273 Calbiochem, MERCK, USA) is an inhibitor of S6K1 (Ki = 20 nM; IC50 = 160 nM). 10 mg of PF–4708671 were fully dissolved in 1 ml DMSO and stored at -80°C for *in vitro* experiments. For *in vivo* assays, PF-4798671 was dissolved in 10% DMSO first and further diluted in 30% PEG400, 0.5% Tween 80 and 5% propylene glycol, to achieve a final DMSO concentration of 1%.

### 2. Cell culture

Three non-small cell lung cancer cell lines were obtained from Type Culture Collection of the Chinese Academy of Sciences, and cultured according to the supplier’s recommendations. They included A549 (adenocarcinoma), NCI-H460 (large cell carcinoma), and SK-MES-1 (squamous cell carcinoma) cells. All cells were maintained in a humidified environment containing 5% CO2 at 37°C.

### 3. Western blot

Proteins were extracted from cells using the phosphorylated protein extraction kit (KeyGEN, Nanjing, China), and concentrations were measured using BCA Protein Assay Reagent (Thermo scientific, Rockford, USA). Equal amounts of protein from various samples were separated by sodium dodecyl sulphate—polyacrylamide gel electrophoresis (SDS-PAGE) and electro-transferred onto polyvinylidene fluoride (PVDF) membranes (Millipore, Billeraica, USA). Then, the membranes were incubated overnight at 4°C with anti-p70S6K R365, anti-p-p70S6K T389, anti-ribosome protein S6 A229 (#BS1568, #BS4440, #BS3610, 1;1000, Bioworld Technology, Nanjing, China), anti-BAD, anti-Caspase3, anti-ERK (#9239P, #96625, #3552S, 1:1000, Cell Signaling Technology), and anti-β-actin (#4970, 1:5000, Cell Signaling Technology) antibodies, respectively. Target proteins were detected using the ChemiDoc XRS system (Bio-Rad, Philadelphia, USA) after exposure to chemiluminescent HRP substrate (Millipore, Billerica, USA). Data were analyzed with the Quantity One 1-D Analysis software (Bio-Rad).

### 4. Cell proliferation assay

Cell proliferation of NSCLC cell lines was measured using Cell Counting kit-8 (CCK-8; Dojindo, Kumamoto, Japan) according to the manufacturer’s protocol. Tumor cells were seeded in 96-well plates at a density of 5×10^3^ per well. After incubation in presence of PF-4708671 (0.1μM, 0.3μM, 1μM, 3μM and 10μM) for 24, 48 and 72 hours, respectively, cell proliferation was assessed. DMSO treated or untreated cells were used as negative controls. Additionally, the cell proliferation marker Ki-67 was examined by immunohistochemistry in nude tumor tissues.

### 5. Cell cycle analysis

For each cell line, approximately 1×10^6^ cells were harvested after treatment with 10μM PF-4708671 for 24h; cell cycle distribution was assessed using Cell Cycle Detection Kit (KeyGEN, Nanjing, China); DNA contents were determined on a FACSCalibur Flow Cytometer (Becton-Dickinson, Franklin Lakes, USA). Data were analyzed using the CellQuest and Modfit software.

### 6. Cell invasion

Cell invasion was evaluated using the Millipore cell invasion assay kit (#ECM550, Millipore, USA) according to the manufacturer’s instructions, after treatment with PF-4708671 at 10μM for 24h. Cell suspensions containing 1×10^6^ cells/ml were seeded onto the upper chamber with medium supplemented with 1% serum. Media containing 20% FBS were added to lower chambers.

### 7. Cell apoptosis

Approximately 5 x 10^5^ cells were harvested after treatment with PF-4708671 at 10μM for 24h, and stained with Annexin V-APC/ 7-AAD Apoptosis Detection Kit (KeyGen, Nanjing, China) according to the manufacturer’s protocol. Fluorescence was measured on a FACSCalibur Flow Cytometer. Annexin V-positive and 7AAD-negative cells considered to be apoptotic. In addition, the TUNEL method was used to determine apoptosis in xenograft tumor tissues.

### 8. *In vivo* effects of PF-4708671 in a nude mouse xenograft model established with H460 cells

H460 cells, which showed the highest growth rate, were selected for *in vivo* experiments. Female nude mice (4 weeks, 18 to 20g) were purchased from Beijing WeiTongLiHua experimental animal technical co., LTD. (China), and housed in the Animal Center of West China Sichuan University, with a 12h light/12h dark cycle. All experiments were carried out in SPF (Special Pathogen Free) conditions. Mice were divided into three groups randomly, and each group contained three mice (n = 3/each group). The end points of observation were as follows: 1) the diameter of tumors > 3cm; 2) transplanted tumors had necrosis and/or decay; 3) natural death. The method of sacrifice at the end of the vivo research was decapitated after anesthesia under the premise of without other mice could see. In Group 1 (H460 group), 1×10^7^ H460 cells were subcutaneously injected into mice. Group 2 (negative control, NC group) animals were injected cells as in Group1; three days later, they were intraperitoneally administered 200μl of solvent mix (1% DMSO, 30% PEG400, 0.5% Tween80 and 5% propylene glycol) daily for 1 week. In Group 3 (H460+PF4708671 group), mice were treated as described for Group 2, with 200μl PF4708671 (50 mg/kg) instead of solvent mix. Tumors sizes were measured daily for more than one week (long diameter = a; short diameter = b), and volumes were derived as follows: V = ab2/2. Tumor inhibition rate = [(control tumor weight—experimental tumor weight)/control tumor weight] x 100%. The study was approved by the ethics committee of West China Hospital.

### 9. Statistical analysis

Data were processed by Microsoft Excel 2010. Using the SPSS 19.0 software, t-test and one way-ANOVA were applied for data analysis. Data are mean±SD, and two-sided *P*<0.05 was considered statistically significant.

## Results

### 1. PF-4708671 inhibits p70S6K and S6 phosphorylation

**Fig 1:**
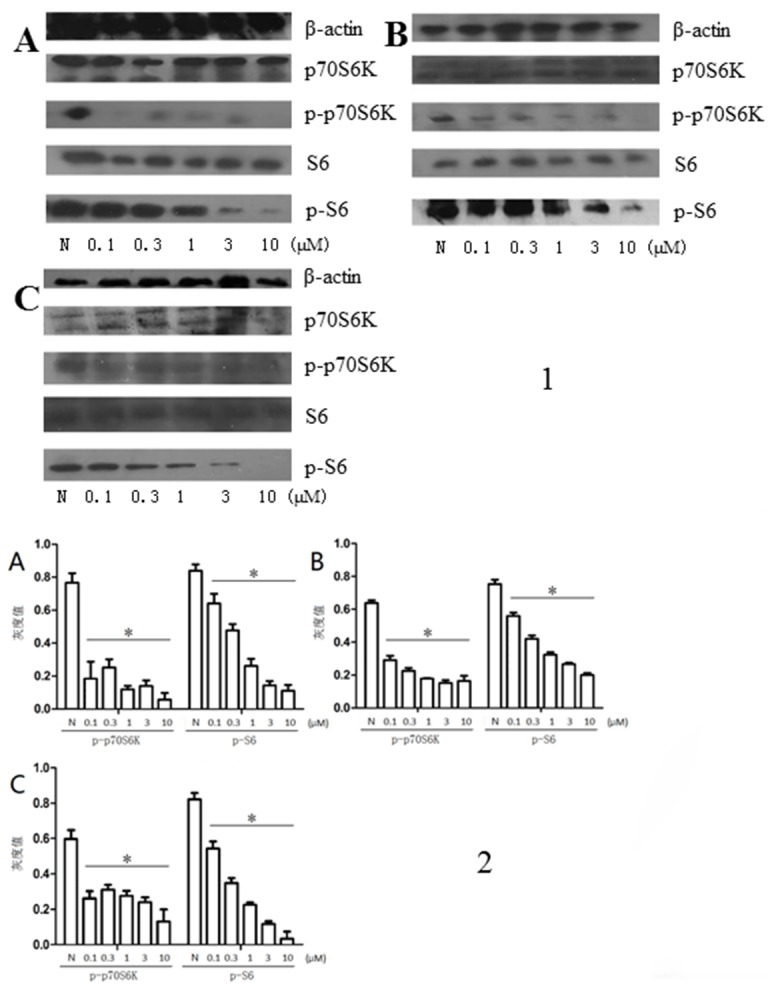
**1. Protein expression levels of p70S6K, p-p70S6K, S6, and p-S6 after treatment with various drug concentrations**. β-actin was used as a loading control. **2. Protein expressions levels of p-p70S6K and p-S6 under different drug concentrations**. *P*<0.05: drug treatment group vs negative control group. N, negative control; A, H460; B, A549; C, SK-MES-1.

Our results revealed that the specific p70S6K inhibitor PF-4708671 significantly reduced the phosphorylation of p70S6K and its downstream S6 at 0.1μM (*P*<0.05); p-S6 expression levels decreased with increasing drug concentrations (*P*<0.05). On the contrary, protein levels of downstream BAD, Caspase3 and ERK were increased after treatment with PF-4708671 with the concentration of 1μM. However, total p70S6K and S6 protein levels had no significant differences between negative control and treatment groups (Fig 1, S1 File).

### 2. PF-4708671 inhibits NSCLC cell proliferation

**Fig 2:**
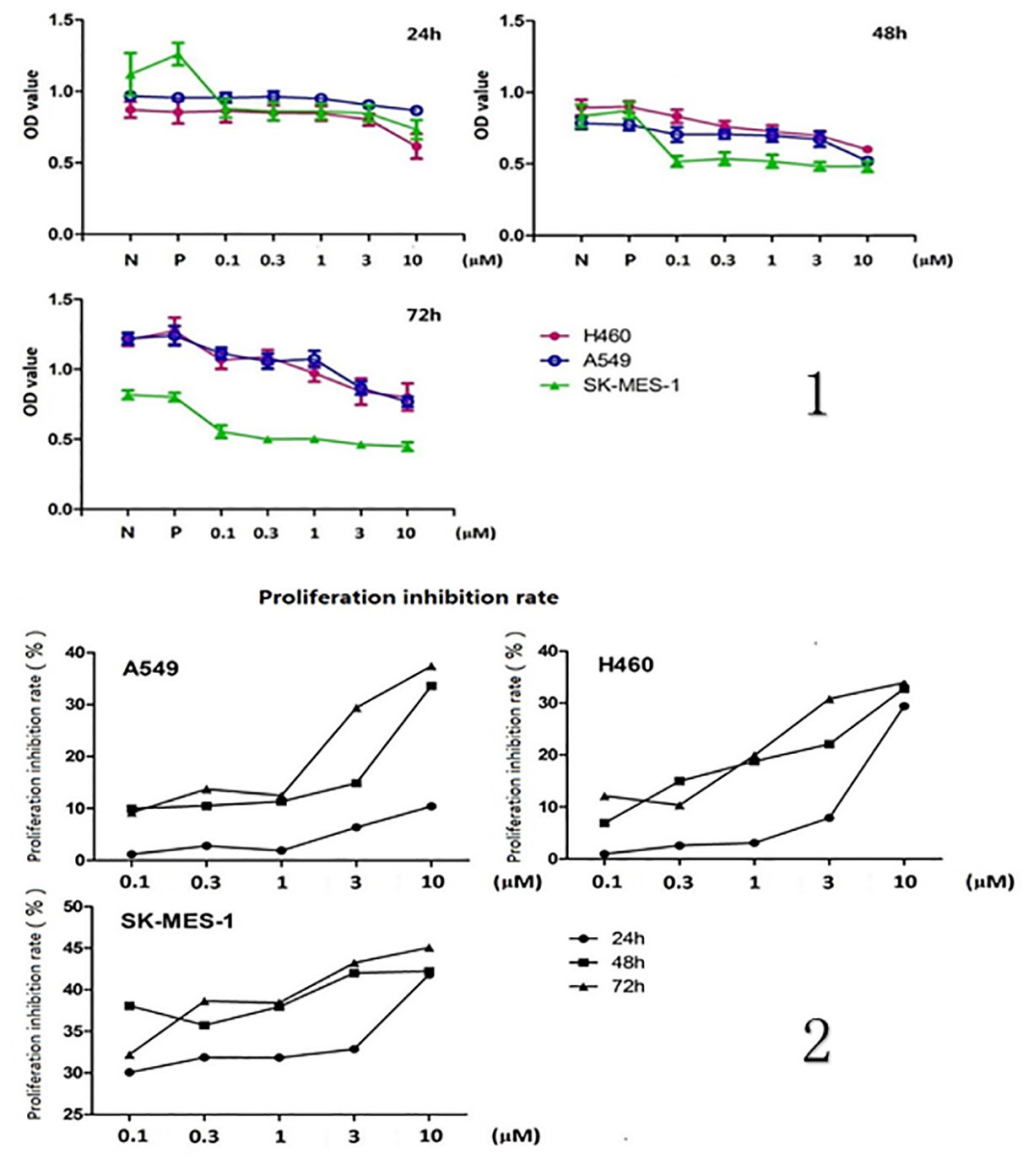
1. Effects of PF-4708671 on NSCLC cell proliferation. 2. Proliferation inhibition rates of NSCLC cell lines. Proliferation inhibition rate = [1 − (experimental group values − blank value)/(negative control group value − blank value)] × 100%.

CCK-8 assay data demonstrated that proliferation abilities of the three NSCLC cell lines were significantly inhibited by PF-4708671. After 24 hours of treatment, PF-4708671 inhibited H460 cell proliferation at 10μM, and A549 and SK-MES-1 cell amounts were significantly reduced at 3μM and 0.1μM, respectively (*P*<0.05). In addition, H460, A549, and SK-MES-1 cell growth rates were significantly inhibited by PF-4708671 at 0.3μM, 0.1μM, and 0.1μM, respectively, 48 hour post treatment (*P*<0.05). All cell lines showed significantly reduced proliferation at 72 hour after treatment with 0.1μM PF-4708671 (*P*<0.05). Significant time- and concentration-dependent inhibition of cell proliferation was observed in all cell lines (*P*<0.05) (Fig 2).

### 3. PF-4708671 affects NSCLC cell cycle

**Fig 3:**
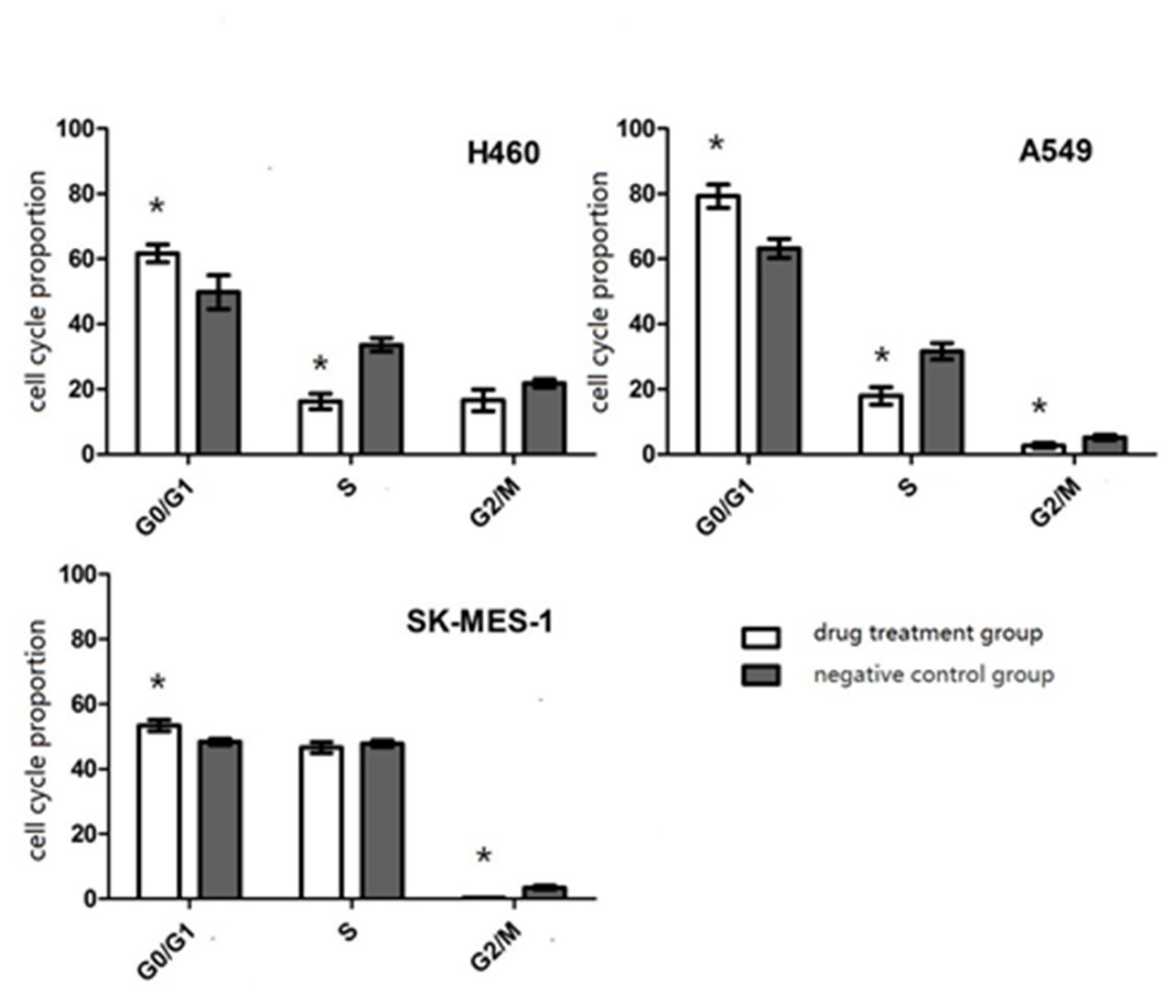
Effects of PF-4708671 on NSCLC cell cycle distribution. (*p<0.05, drug treatment group vs negative control group).

After DNA staining by PI, flow cytometry analysis showed cell cycle arrest in G0/G1 phase for all three NSCLC cell lines (*P*<0.05). Meanwhile, the proportions of cells in S and G2/M phases were significantly decreased in A549 cells (*P*<0.05). In addition, reduced amounts of S phase (H460) and G2/M phase (SK-MES) cells were also detected (*P*<0.05) (Fig 3).

### 4. PF-4708671 significantly inhibits cell invasion

**Fig 4:**
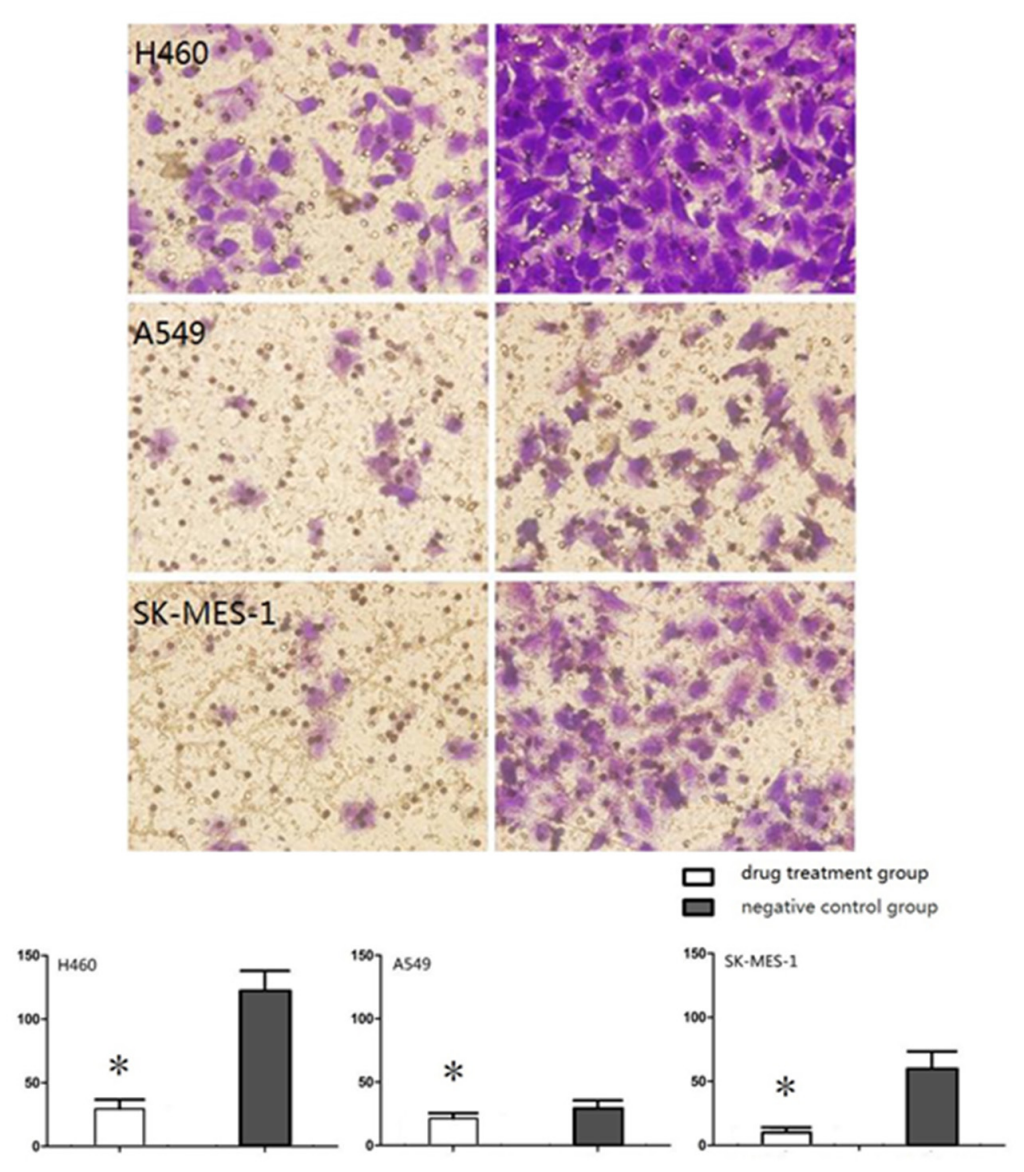
Effects of PF-4708671 on NSCLC cell invasion.

PF-4708671 inhibited invasion ability in the three NSCLC cell lines assessed. Indeed, average numbers of cells passing through the extracellular matrix gel were significantly lower in treatment groups than in negative controls (*P*<0.05), with invasion inhibition rates of 75.90%, 46.23% and 82.73%, for H460, A549 and SK-MES-1 cells, respectively (Fig 4).

### 5. PF-4708671 increases NSCLC cell apoptosis

**Fig 5:**
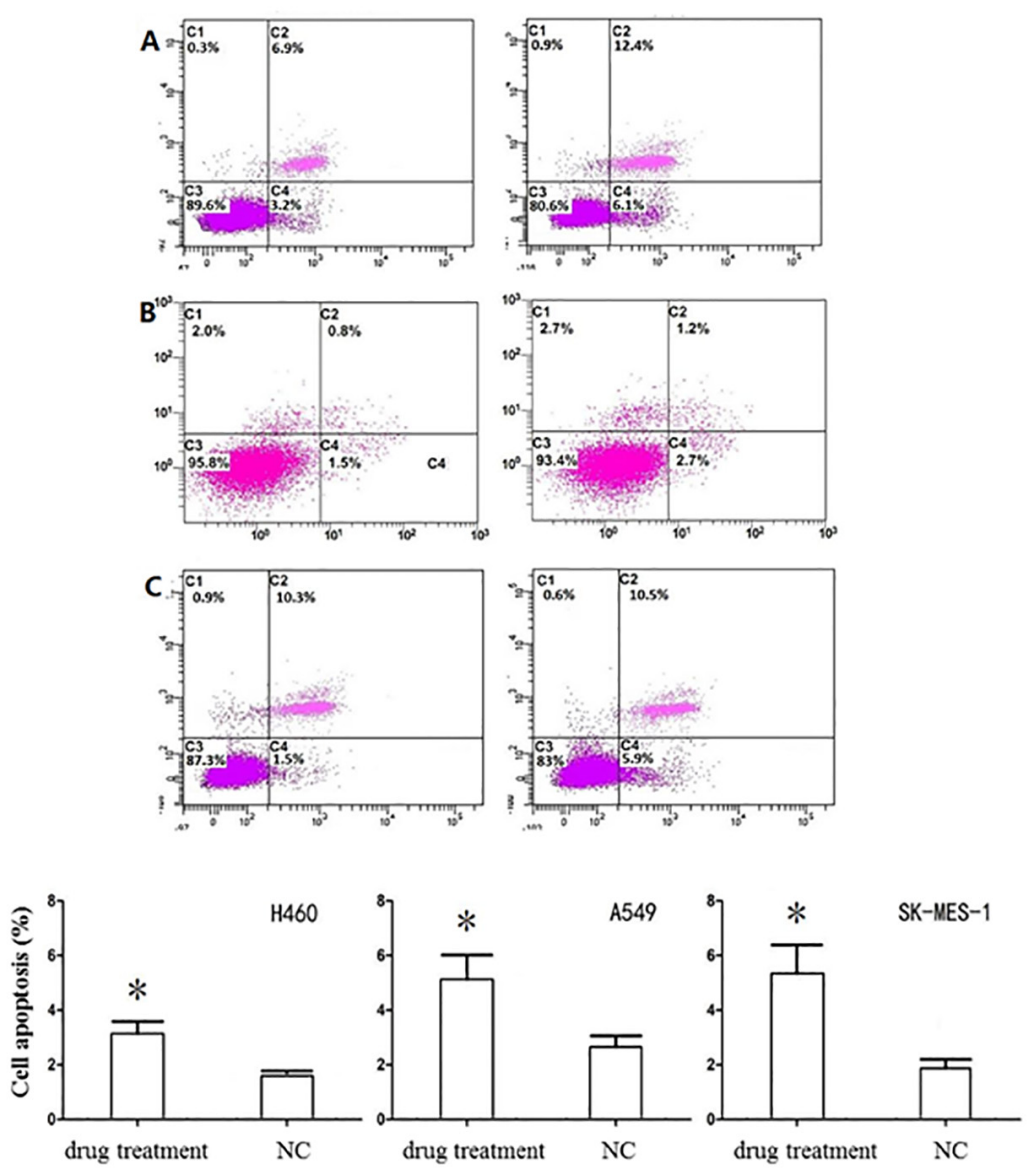
Effects of PF-4708671 on NSCLC cell apoptosis.

PF-4708671 showed limited effect on apoptosis in the three NSCLC cell lines, although statistically significant differences were obtained (*P*<0.05). Compared with negative control groups, apoptosis rates for H460, A549 and SK-MES-1 cell lines were increased by 1.537%, 2.483% and 3.475%, respectively (*P*<0.05) (Fig 5).

### 6. PF-4708671 inhibits tumorigenesis *in vivo*

**Fig 6:**
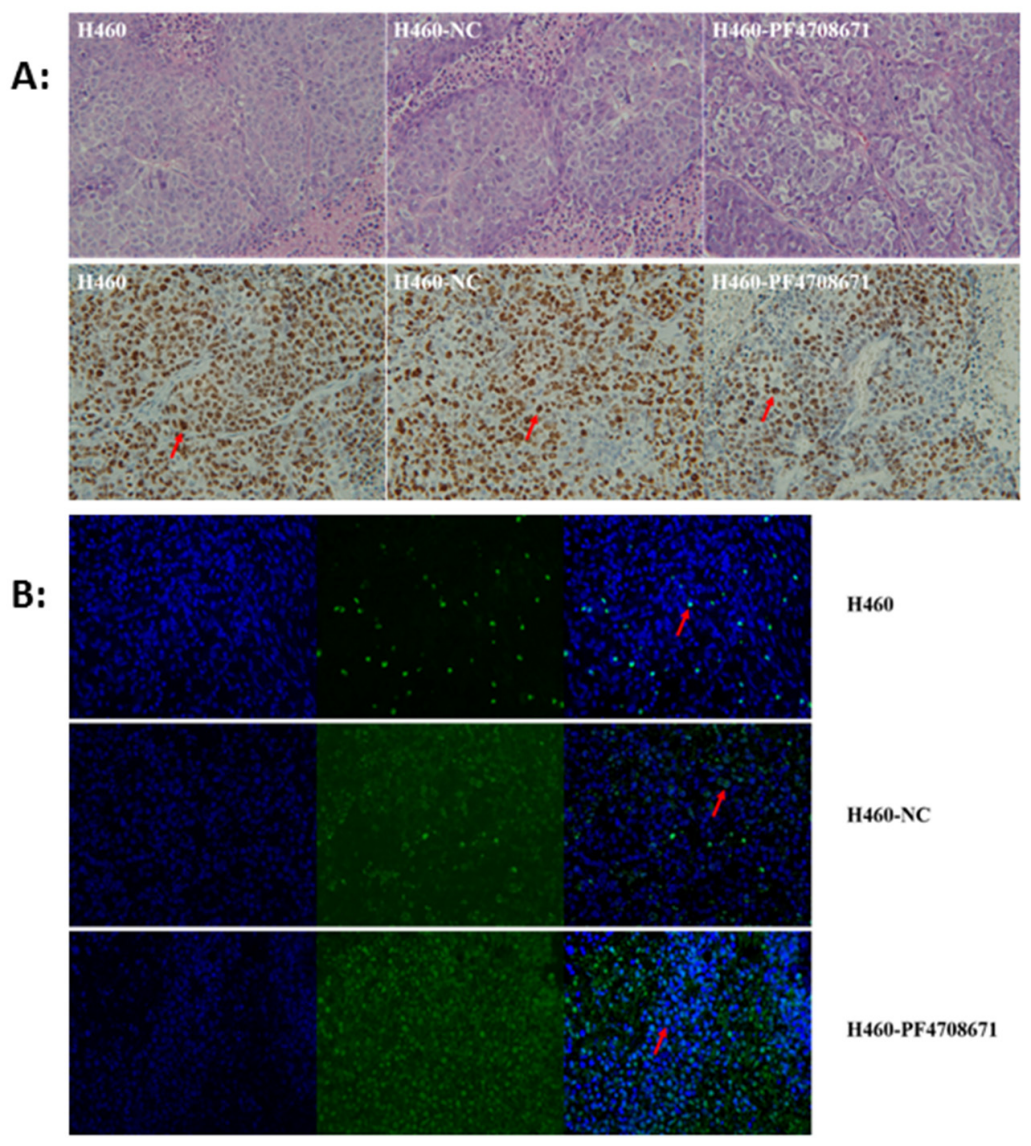
A. Hematoxylin-eosin (HE) staining and Ki-67 expression in various groups. B. Expression of terminal deoxynucleotidyltransferase-mediated dUTP nick end labeling (TUNEL). Arrows show positively stained cells. Original magnification, ×400.

All transplanted tumors were grown out successfully in each mouse, and all mice have no signs of miscomfort and/or suffering until we put them to death. The trends of tumor volumes in nude mice for each group are summarized in Table A in S1 File and S2 Fig. Animals treated with the p70S6K specific inhibitor PF470867 showed overtly inhibited tumor growth compared with Group 1 (H460) and Group 2 (NC) (all *P*<0.05). As shown in Table B in S1 File and S3 Fig, weights and volumes of the excised tumors were lower after treatment with PF470867 compared with control values (*P*<0.05). Furthermore, cell proliferation, as expressed by Ki-67, was significantly reduced in the H460+PF4708671 group compared with values obtained for both control groups. Finally, TUNEL staining demonstrated significantly higher apoptotic rate for tumor cells in Group 3 compared with control (Groups 1 and 2) values (Fig 6).

## Discussion

P70S6K participates in tumorigenicity and cancer progression, but the mechanism underlying its effect is not completely understood. Studies assessing p70S6K in relation to NSCLC are scarce; we previously found that p70S6K overexpression promotes NSCLC cell proliferation, inhibits cell apoptosis and enhances invasion ability. To further understand the role of p70S6K inhibitors in non-small cell lung cancer, the present study assessed three NSCLC cell lines (A549, SK-MES-1, and NCI-H460) treated with PF-4708671, and evaluated the changes of malignant phenotypes such as proliferation, invasion and apoptosis evasion.

As shown above, the p70S6K inhibitor PF-4708671 significantly inhibited the activation of p70S6K and its key downstream effector S6, in a concentration dependent manner. Furthermore, the protein levels of downstream BAD; Caspase3 and ERK, which affecting apoptosis ability of cells, were increased after treatment with PF-4708671. This proved that PF-4708671 might affect the NSCLC cell lines’ survival by regulating relevant factors of apoptosis. In addition, NSCLC cell proliferation was time and concentration dependently inhibited by PF-4708671, corroborating previous reports [15–17]. Thus, we proposed that p70S6K regulation is associated with cell apoptosis. The differences in minimum effective drug concentrations for the NSCLC cell lines assessed may be due to their distinct p70S6K levels and growth rates: we found growth inhibition rate of 30 to 40%.

We hypothesized that by regulating the cell cycle, PF-4708671 may inhibit cell proliferation. Recent studies showed that S6K1 and p70S6K are critical for G1 arrest [26–28]. Consistently, we found that PF-4708671 affected cell cycle distribution in the three NSCLC cell lines assessed, significantly delaying cell cycle progression in G0/G1 phase. Another important factor affecting cell proliferation is apoptosis; a phase I trial of LY2584702 evaluating advanced solid tumor patients showed no overt anti-tumor effect by this drug [25]. As shown above, PF-4708671 had limited effect on cell apoptosis in NSCLC cells, with an apoptosis rate close to 3% in vitro. Therefore, we assumed that p70S6K pathways may not be involved in cell apoptosis. However, this study also demonstrated that PF-4708671 inhibits NSCLC tumorigenesis *in vivo*.

p70S6K overexpression was shown to be associated with aggressive disease and poor prognosis in breast cancer [29]. Our previous study showed p70S6K overexpression promotes cell invasion *in vitro*. Interestingly, PF-4708671 could inhibit the invasion ability of all NSCLC cell lines assessed in this study. In contrast, studies in NSCLC patients showed that p-p70S6K expression is not associated with lymph node metastasis and cancer stage [18–29]. Further studies are needed to confirm the role of p70S6K in NSCLC invasion and metastasis *in vivo*.

However, there still had some limitations in our research. First of all, although all cells were NSCLC cell lines, the sources were different. So our results could not be completely consistent because there might be different mechanisms among different genetic backgrounds. Moreover, quantity was not big enough for experiments of nude mice in vivo. Therefore, the results need further in-depth research to confirm.

In conclusion, our study demonstrated that the p70S6K inhibitor PF-4708671 could affect cell cycle distribution, inhibiting cell proliferation, apoptosis and invasion in NSCLC cells. Inhibiting the p70S6K-S6 axis resulted in potent anti-tumor activity. Therefore, combination therapy with p70S6K inhibitor and chemotherapy represents a promising new strategy for NSCLC treatment.

## Supporting Information

S1 Fig Protein expression levels of BAD, Caspase3 and ERK after treatment with PF-4708671. (TIF)

S2 Fig Tumor volume growth trend in each group of nude mice (mm^3^). (TIF)

S3 Fig Comparison of tumor sizes among groups. (TIF)

S1 FileTable A. Tumor volume growth of each group in nude mice ($\bar{x}$, n = 3/mm^3^). Table B. Tumor xenografts weight of each group ($\bar{x}\pm s$).(DOCX)
