# Supplementary figures and images for: The p70S6K Specific Inhibitor PF-4708671 Impedes Non-Small Cell Lung Cancer Growth
Source: PLoS One. 2016 Jan 15;11(1):e0147185. doi: 10.1371/journal.pone.0147185 (PMC4714881; doi:10.1371/journal.pone.0147185)

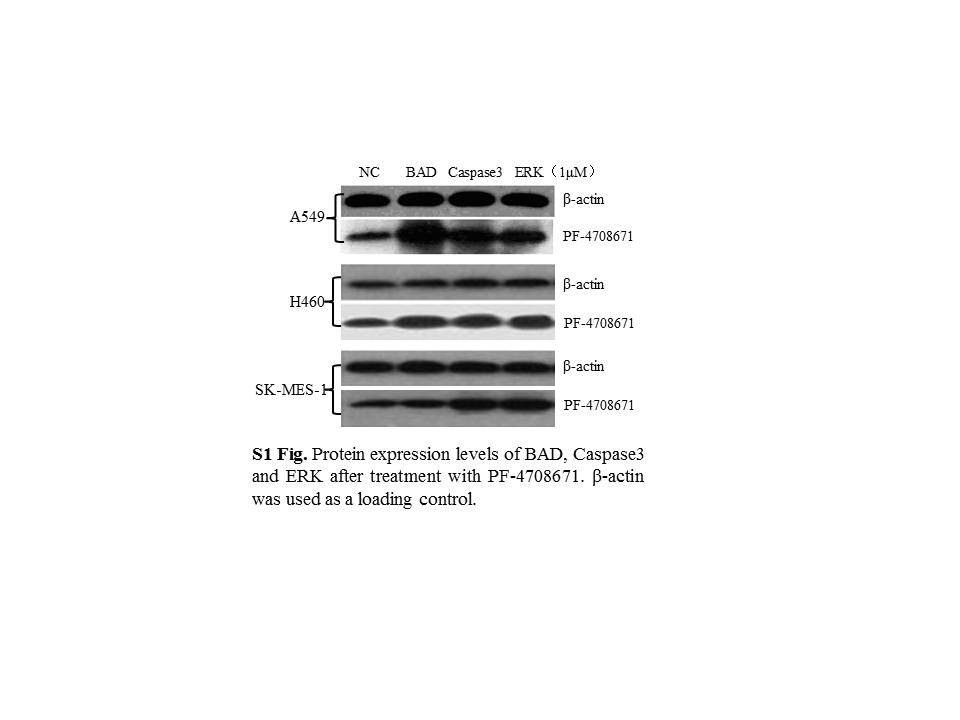

Supplement: S1 Fig — (TIF) [file pone.0147185.s001.tif]

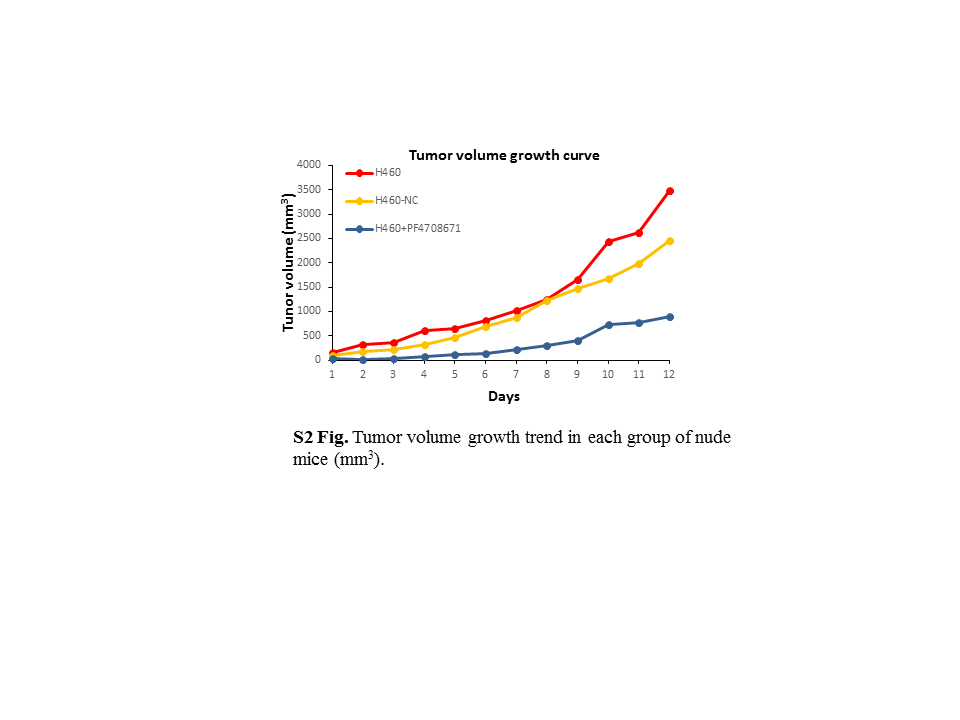

Supplement: S2 Fig — (TIF) [file pone.0147185.s002.tif]

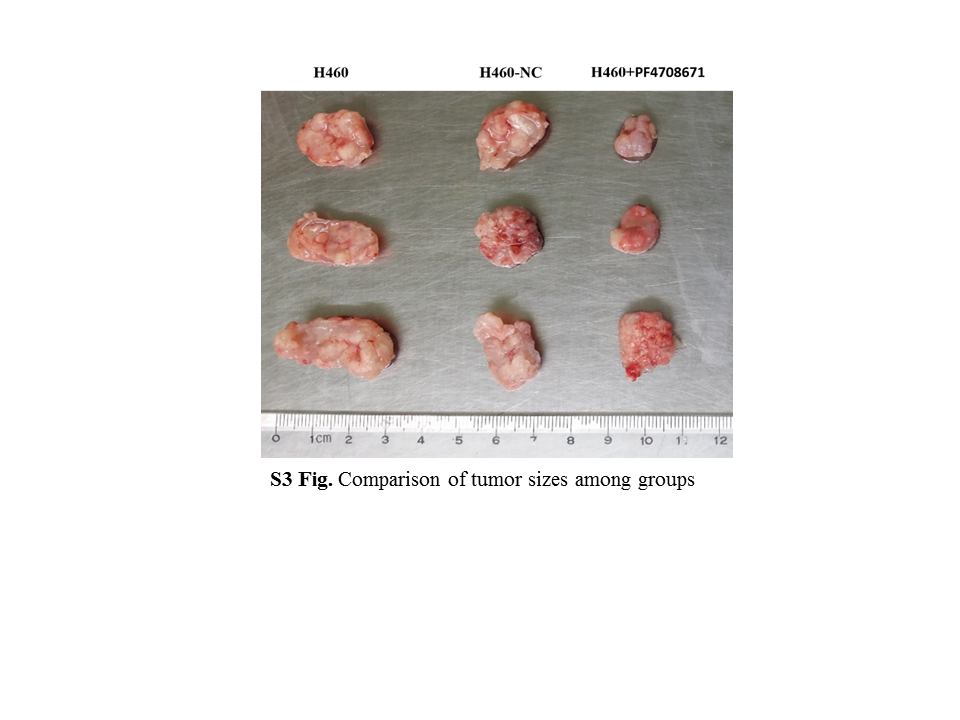

Supplement: S3 Fig — (TIF) [file pone.0147185.s003.tif]
