# Supplementary material for: The p70S6K Specific Inhibitor PF-4708671 Impedes Non-Small Cell Lung Cancer Growth
Source: PLoS One. 2016 Jan 15;11(1):e0147185. doi: 10.1371/journal.pone.0147185 (PMC4714881; doi:10.1371/journal.pone.0147185)
Supplement: S1 File — (DOCX) [file pone.0147185.s004.docx]

**Supplementary table-A Tumor volume growth of each group in nude mice ($\bar{x}$，n=3/mm^3^)**

| Groups | Day 1 | Day 2 | Day 3 | Day 4 | Day 5 | Day 6 | Day 7 | Day 8 | Day 9 | Day 10 | Day 11 | Day 12 |
| --- | --- | --- | --- | --- | --- | --- | --- | --- | --- | --- | --- | --- |
| H460 | 154.00 | 310.67 | 350.33 | 603.67 | 654.33 | 803.33 | 1025.33 | 1253.33 | 1650.00 | 2427.00 | 2628.00 | 3477.00 |
| H460-NC | 93.33 | 174.50 | 226.00 | 327.50 | 472.00 | 694.00 | 874.67 | 1223.67 | 1467.50 | 1668.00 | 1983.67 | 2450.00 |
| H460+PF4708671 | 28.00 ^ab^ | 18.00 ^ab^ | 25.50 ^ab^ | 74.00 ^ab^ | 115.00 ^ab^ | 133.33 ^ab^ | 226.00 ^ab^ | 306.00 ^ab^ | 400.67 ^ab^ | 724.33 ^ab^ | 775.00 ^ab^ | 885.00 ^ab^ |

^a^ p<0.05 H460+PF4708671 vs H460, ^b^ p<0.05 H460+PF4708671 vs H460-NC

**Supplementary table-B Tumor xenografts weight of each group ($\bar{x}\pm s$)**

| **Groups** | **Weights （n=3/mg）** |
| --- | --- |
| H460 | 2733.33±513.16 |
| H460-NC | 2366.66±57.73 |
| H460+PF4708671 | 1900.00±360.55^ab^ |

^a^ p<0.05 H460+PF4708671 vs H460, ^b^ p<0.05 H460+PF4708671 vs H460-NC
